# The light side of the force

**DOI:** 10.7554/eLife.14274

**Published:** 2016-02-23

**Authors:** Aakash Basu, Taekjip Ha

**Affiliations:** 1 Department of Biophysics and Biophysical Chemistry, Johns Hopkins University, Baltimore, United States; 2 Department of Biophysics and Biophysical Chemistry, the Department of Biomedical Engineering and the Howard Hughes Medical Institute, Johns Hopkins University, Baltimore, United Statestjha@jhu.edu

**Keywords:** optical trap, smFRET, single molecule biophysics, TPP, riboswitch, None

## Abstract

A combination of two single-molecule techniques has revealed new tertiary interactions in the TPP riboswitch.

**Related research article** Duesterberg VK, Fischer-Hwang IT, Perez CF, Hogan DW, Block SM. 2015. Observation of long-range tertiary interactions during ligand binding by the TPP riboswitch aptamer. *eLife* **4**:e12362. doi: 10.7554/eLife.12362

**Image** Schematic of the experimental setup used to study the TPP riboswitch.

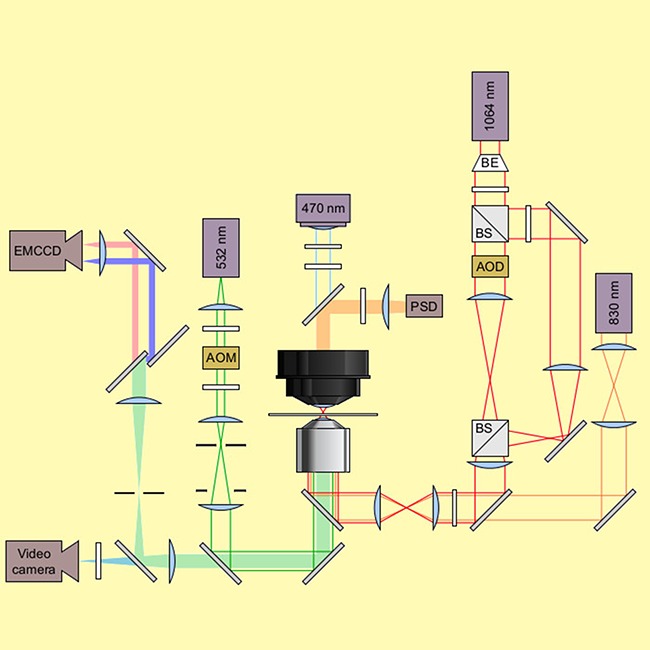

Single-molecule techniques are widely used to investigate how the structure of a molecule changes over time. Such measurements have advantages over standard biochemical experiments, which can only record average values measured over a large number of molecules, and traditional structural biology techniques that cannot gather dynamic information. However, a drawback of single-molecule techniques is that they typically involve measuring how just a few structural parameters – often only one, such as the end-to-end length of the molecule – vary over time. Thus, a growing trend in the field is to use hybrid instruments that combine different single-molecule techniques, and so allow more than one parameter to be observed simultaneously (Ha, 2014).

Riboswitches are regions in the non-coding regions of messenger RNA (mRNA) molecules that control the expression of the mRNA by sensing and binding to specific small 'ligand' molecules. Riboswitches comprise an aptamer region that undergoes structural transitions when a ligand binds to it, and an expression platform that regulates the expression of the mRNA in response to these shape changes. Now, in eLife, Steven Block and co-workers from Stanford University – Van Duesterberg, Irena Fischer-Hwang, Christian Perez and Daniel Hogan – combine two single-molecule techniques to investigate the structural changes that occur in the thiamine pyrophosphate (TPP) riboswitch as it unfolds and binds to TPP and other ligands (Duesterberg et al., 2015).

Most riboswitches are present only in bacteria, but the TPP riboswitch is present in all kingdoms of life. When bound to a molecule of TPP, the structure of this riboswitch resembles an inverted ‘h’ (Figure 1), with TPP docked in the region between the two 'sensor arms' of the aptamer.

**Figure 1.**
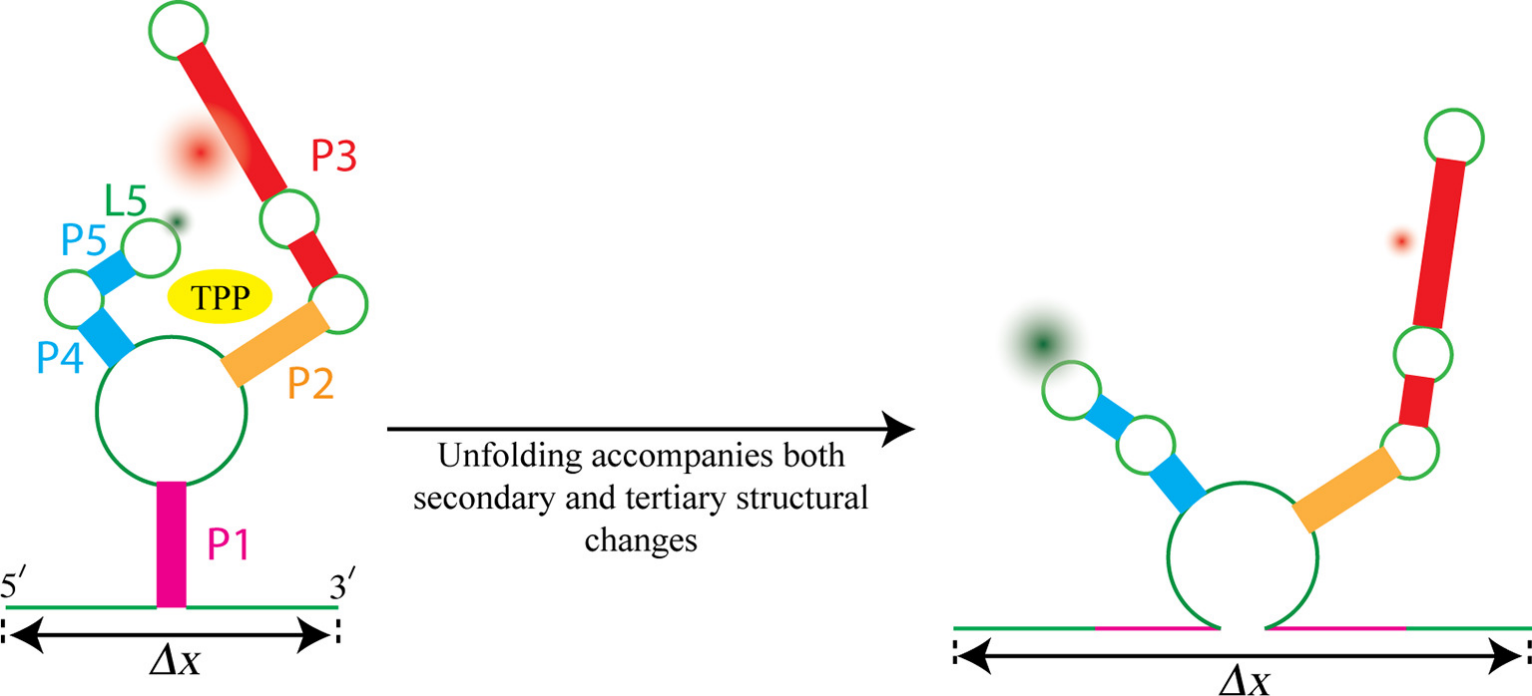
Watching the TPP riboswitch unfold. The ligand TPP (yellow) binds to the riboswitch in the region between the two sensor arms of its aptamer, and also makes contact with each arm. There is also a long-range tertiary interaction between the loop L5 at the tip of one arm and the helix P3 in the other arm. Duesterberg, Fischer-Hwang, Perez et al. used optical trapping to apply a pulling force to the ends of the molecule and to measure the overall end-to-end extension (Δ*x*). Simultaneously, they used smFRET to measure the separation between fluorophores (green and red fuzzy circles) placed on L5 and P3. Mechanical unfolding and the state of ligand binding affect both secondary structures and tertiary interactions. Secondary structural changes, such as unzipping one or more helices (helix P1 in this example), cause a corresponding change in Δ*x*. Changes in long-range tertiary structure, such as the two sensor arms docking with each other, does not affect Δ*x*, but cause a corresponding change in FRET efficiency.

Force-extension curves can be obtained by stretching individual riboswitches and measuring the end-to-end length of the molecule (Greenleaf et al., 2008). This technique has been used to monitor the mechanical unfolding of secondary structures in the TPP riboswitch, such as the various helixes that make up the aptamer (Anthony et al., 2012). Depending on how the TPP molecule is bound to the riboswitch (it can be bound strongly, weakly or not at all), these secondary structures unfold at different values of pulling force, thus producing distinct force-extension curves. However, these curves are typically insensitive to long-range tertiary interactions, such as the interactions that occur between the L5 structural loop at the tip of one sensor arm and the P3 helix at the end of the other arm (Figure 1).

Additionally, the pattern of secondary structure unfolding of the weakly bound form of the riboswitch does not change when TPP is replaced with thiamine monophosphate (TMP, which contains one phosphate group, rather than the two found in TPP) or thiamine (which contains no phosphate groups). This leaves open the possibility that the phosphates alter tertiary interactions, as these changes are undetectable in force-extension curve measurements.

A technique called smFRET has been used extensively to investigate how tertiary structures change in large molecular complexes and, more recently, in riboswitches (Lemay et al., 2006). Now, Duesterberg, Fischer-Hwang, Perez et al. have used a combined force-smFRET approach (Hohng et al., 2007; Tarsa et al., 2007) to stretch the TPP riboswitch complex while simultaneously observing both the end-to-end extension and changes in long-range tertiary interactions. smFRET measures the latter by monitoring the energy transfer between two fluorophores placed on the P3 helix and the L5 loop; the smFRET signal depends on the distance between these structural elements.

Duesterberg et al. tested TPP riboswitches that were weakly bound to one of TPP, TMP or thiamine. All three ligands showed the same pattern of force-induced unfolding of secondary structures. However, the smFRET results indicated that P3 and L5 were further apart when the riboswitch was bound to TMP or thiamine. Thus, each additional phosphate group seems to draw the sensor arms of the riboswitch closer together during ligand binding.

Further, although the two arms are more separated in the presence of bound TMP or thiamine, in all cases there was some variability in the separation distance between the arms. However, this variability is greater in the TPP-bound case. Duesterberg et al. hypothesize that the flexibility of the riboswitch’s structure when weakly bound to TPP might be essential for proper docking of the helix arms and the subsequent transition of the aptamer to the strongly bound configuration.

Both smFRET and force-extension curves have widely been used to study the folding of large biological molecules. However, this work by Duesterberg et al. represents the first example of combining the two in order to simultaneously report on secondary and tertiary structural changes during folding and ligand binding. Large-scale tertiary structural transitions are ubiquitous in biology. Applying similar approaches to the wide body of proteins and RNAs whose folding characteristics have been studied by force-extension curves alone will greatly enhance our understanding of the overall structural transitions essential for their biological functions.

The relative ease of implementation of smFRET detection has made it a technique of choice for combining with other single-molecule techniques. For instance, FRET has recently been combined with patch clamping in order to correlate structural transitions in ion channels with the flow of ionic current (Sasmal and Lu, 2014). Combining traditional single-molecule workhorses, as well as pairing single-molecule techniques with other recent advances in coarse-grained modeling and genomic techniques, will likely serve the critical need to increase the number of observable parameters available when studying molecules one at a time.
